# Systems toxicology approaches enable mechanistic comparison of spontaneous and cigarette smoke-related lung tumor development in the A/J mouse model

**DOI:** 10.2478/intox-2014-0010

**Published:** 2014-11-15

**Authors:** Karsta Luettich, Yang Xiang, Anita Iskandar, Alain Sewer, Florian Martin, Marja Talikka, Patrick Vanscheeuwijck, An Berges, Emilija Veljkovic, Ignacio Gonzalez-Suarez, Walter Schlage, Julia Hoeng, Manuel Peitsch

**Affiliations:** 1 Biological Systems Research, Philip Morris International Research and Development, CH-2000 Neuchâtel, Switzerland; 2 Philip Morris Research Laboratories bvba, 3001 Leuven, Belgium; # Both authors contributed equally to this manuscript

**Keywords:** A/J mouse, lung tumorigenesis, cigarette smoke exposure, gene expression, microRNA (miRNA) expression

## Abstract

The A/J mouse is highly susceptible to lung tumor induction and has been widely used as a screening model in carcinogenicity testing and chemoprevention studies. However, the A/J mouse model has several disadvantages. Most notably, it develops lung tumors spontaneously. Moreover, there is a considerable gap in our understanding of the underlying mechanisms of pulmonary chemical carcinogenesis in the A/J mouse. Therefore, we examined the differences between spontaneous and cigarette smoke-related lung tumors in the A/J mouse model using mRNA and microRNA (miRNA) profiling. Male A/J mice were exposed whole-body to mainstream cigarette smoke (MS) for 18 months. Gene expression interaction term analysis of lung tumors and surrounding non-tumorous parenchyma samples from animals that were exposed to either 300 mg/m^3^ MS or sham-exposed to fresh air indicated significant differential expression of 296 genes. Ingenuity Pathway Analysis^®^ (IPA^®^) indicated an overall suppression of the humoral immune response, which was accompanied by a disruption of sphingolipid and glycosaminoglycan metabolism and a deregulation of potentially oncogenic miRNA in tumors of MS-exposed A/J mice. Thus, we propose that MS exposure leads to severe perturbations in pathways essential for tumor recognition by the immune system, thereby potentiating the ability of tumor cells to escape from immune surveillance. Further, exposure to MS appeared to affect expression of miRNA, which have previously been implicated in carcinogenesis and are thought to contribute to tumor progression. Finally, we identified a 50-gene expression signature and show its utility in distinguishing between cigarette smoke-related and spontaneous lung tumors.

## Introduction

Mouse models have been employed to study the carcinogenicity of chemical compounds including cigarette smoke and to infer underlying mechanisms of lung tumor development in humans (Meuwissen & Berns, 2005). Different strains of mice display markedly varied sensitivity to lung tumor development (Gordon & Bosland, 2009). For example, strain A mice spontaneously develop pulmonary tumors with age and in response to treatment with chemical carcinogens or cigarette smoke. In this model, *K-ras* oncogene activation is associated with enhanced risk of lung tumor susceptibility, illustrated by presentation of pulmonary adenoma (Chen *et al.*, 1994). However, the A/J mouse model has other disadvantages including the fact that there is only a small increase in the number of mostly benign lung tumors after chemical exposure and a shallow dose-response curve (Coggins, 2010).

In the past, a number of A/J mouse studies with cigarette smoke were performed in which mice were exposed to either mainstream cigarette smoke (MS) or a combination of MS and sidestream cigarette smoke (SS), which is used as an experimental surrogate for environmental tobacco smoke exposure (Coggins, 2010). Although a considerable number of studies failed to produce significant increases of either tumor incidence or multiplicity in A/J mice that were chronically exposed to cigarette smoke, other studies, including those that were performed by our group, successfully demonstrated significantly more lung tumors following exposure to MS or the combination of MS and SS relative to sham (Stinn *et al.*, 2010; Stinn *et al.*, 2005). Moreover, an 18-month MS exposure regimen has been shown to be sufficient in eliciting a concentration-dependent lung tumor response (Stinn *et al.*, 2013a). This is in contrast to the previously reported exposure regimen that included a 5-month exposure followed by a 4-month post-inhalation period which was thought to be required and optimal for the full development of cigarette smoke-associated pulmonary lesions in this mouse model (Witschi, 2005).

Although some evidence supports cigarette-smoke-induced inflammation as a major contributor to lung tumor formation in the A/J mouse (Bauer & Rondini, 2009), there is a gap in our understanding of the underlying mechanisms of A/J pulmonary carcinogenesis related to proven and suspected lung carcinogens. One major limiting factor in elucidating the molecular events leading to tumor formation in the context of cigarette smoking is the inherently high susceptibility to spontaneous lung tumor development in air-exposed or sham A/J mice (Chen *et al.*, 1994). We therefore conducted this study to examine the differences between spontaneous and cigarette smoke-related lung tumors in the A/J mouse model of lung tumorigenesis using gene expression and miRNA profiling and sought to identify possible discriminators between these two tumor entities.

## Materials and methods

### Animal Experiments

The current study was a sub-study of an A/J mouse validation study, and its design has been previously described in detail (Stinn *et al.*, 2013a; Stinn *et al.*, 2013b). Briefly, male A/J mice aged between 8 and 12 weeks received whole-body exposure to MS at concentrations of 75, 150, and 300 mg/m^3^ of total particulate matter (TPM) (animal groups MS-75, MS-150, and MS-300, respectively) from the 3R4F reference cigarette (University of Kentucky, Lexington, KY, USA) for 6 h/day and 5 days/week for 18 months. A group of age-matched male mice exposed to air was included as sham control. A 2-day post-inhalation period was added to allow for the down-regulation of most of the acute effects of MS exposure on gene expression, thus enabling the examination of longer-term effects that show the characteristics of the tumorigenic process.

All animal experimental procedures were in conformity with the American Association for Laboratory Animal Science (AALAS) Policy on the Humane Care and Use of Laboratory Animals (AAALAC, 1991) and were approved by the Institutional Animal Care and Use Committee (IACUC, Leuven, Belgium).

### Histopathology

Histopathologic evaluation was conducted on mice exposed to air (sham control, N=27) and to MS of the 3R4F cigarette smoke, (N=33, 37, and 34 mice of the MS-75, MS-150 and MS-300 groups, respectively) by a certified pathologist, who was blinded to the individual treatment of the animals, as reported previously (Stinn *et al.*, 2013a).

### Gene expression analysis

Gene expression analysis was performed on pairs of matched tumor nodule (T) and surrounding, non-tumorous parenchyma (P) tissues from the cigarette smoke-exposed mice and sham controls as described previously (Stinn *et al.*, 2013a). Frozen sections (20 µm) from whole lung tissue were placed on sterilized glass slides and stained with cresyl violet (Sigma, Taufkirchen, Germany). Lung tumor and parenchyma tissues were collected from these slides using laser capture microdissection (PALM^®^ Microbeam, Zeiss, Jena, Germany). One slide was preserved for the histopathologic characterization of the tumor. The tissues were immediately lysed with Qiazol lysis buffer (Qiagen, Hilden, Germany), and RNA was isolated using the miRNeasy Mini Kit (Qiagen) according to the manufacturer's protocol. All RNA samples had an RNA integrity number (RIN) ≥6.0. Transcriptomic analysis was performed using the Affymetrix Gene Chip^®^ Mouse Genome 430 v2.0 (Affymetrix, Santa Clara, CA, USA). After synthesis of double-stranded cDNA, biotinylated complementary RNA (cRNA) was generated and fragmented. The degree of fragmentation and the length distribution of this fragmented biotinylated cRNA was checked by capillary electrophoresis using the Agilent 2100 Bioanalyzer (Agilent Technologies, Waldbronn, Germany). Ten micrograms of biotinylated fragmented cRNA was hybridized to the GeneChip^®^ for 16 h at 60 rpm in a 45°C GeneChip^®^ Hybridization Oven 640 (Affymetrix). The arrays were washed and stained on the GeneChip^®^ Fluidics Station 450 (Affymetrix), followed by antibody signal amplification, washing, and staining on using streptavidin R-phycoerythrin. The arrays were scanned using the Agilent Gene Array Scanner 3000 7G. Scanned image files were visually inspected for artifacts and then submitted to analysis.

### MicroRNA analysis

The same preparations of total RNA were used for miRNA expression profiling. The dual-channel Exiqon miRCURY LNA™ platform was used for hybridization, which was performed directly by Exiqon (Vedbaek, Denmark). The FirstChoice Human Total RNA Survey Panel (Ambion, Austin, TX, USA) was used for the reference RNA. Total RNA samples (500 ng) and reference RNA samples (100ng) were labeled with Hy3™ and Hy5™ fluorescent labels, respectively, using the miRCURY™ Hy3/Hy5 Power labeling kit (Exiqon, Vedbaek, Denmark). Each total RNA sample was mixed with a reference RNA sample and hybridized in a randomized order to the miRCURY LNA™ miRNA arrays (version 11), following a common reference design where each experimental sample is hybridized against a common reference sample. The arrays were annotated for the probes targeting all miRNAs for human, mouse, or rat registered in the miRBASE miRNA registry version 16. The hybridization was performed using a Tecan HS4800 hybridization station (Tecan, Vienna, Austria). Following hybridization and processing, the arrays were scanned using the Agilent G2565BA Microarray Scanner System (Agilent Technologies, Santa Clara, CA) and quantification of the signals was performed using ImaGene 9.0 software (BioDiscovery, Hawthorne, CA, USA). Raw data were provided in an ImaGene text file format.

### Statistical and computational analysis

#### Gene expression data processing

The quality of the Affymetrix.CEL files was checked by utilizing the R packages *affy*, *gcrma*, and *affyPLM* (Bolstad *et al.*, 2003; Gautier *et al.*, 2004; Wu *et al.*, 2005). Normalized unscaled standard error (NUSE) and relative log expression (RLE) box plots were generated to identify potential outliers. Microarray expression values were generated from the.CEL files using background correction, quantile normalization, and median polish summarization.

#### Gene expression interaction analysis

Surrounding, non-tumorous parenchyma (P) and matched tumor tissue (T) samples from 2 sham controls, 2 MS-75, 5 MS-150 and 5 MS-300 animals were available for gene expression analysis (Table 1). Interaction effects between tissue type (T or P) and cigarette smoke concentration on gene expression were determined using the following linear model:

*GxP =β*_*0*_ *+β*_*1*_ *×CeT+β*_*2*_ *×doL+β*_*3*_ *×doM+β*_*4*_ *×doH+β*_*5*_ *×CeT:doL+β*_*6*_ *×CeT:doM+β*_*7*_ *×CeT:doH+ɛ*

**Table 1:** Summary of tissue samples used for gene expression analysis.

Tissue	Number of Mice			
	Sham	MS-75	MS-150	MS-300
Parenchyma	2	2	5	5
Tumor	8	8	6	9

with L, M, and H denoting MS-75 (L), MS-150 (M), and MS-300 (H) groups, respectively, and *ɛ* is the error term. The term *CeT* represents the effects of tumor tissue, and the terms *doL*, *doM* and *doH* indicate the effects of the different MS concentrations. The interaction coefficient *CeT:doH* describes the difference in gene expression between tumor and parenchyma of the mice from the MS-300 group as compared to the sham controls. For the MS-75 and MS-150 groups, the interaction terms were defined similarly. The cutoff for the false-discovery rate (FDR) was 0.01.

Genes that were significantly altered under the interaction analysis were further analyzed with respect to their functions and roles in signaling pathways using Ingenuity Pathway Analysis^®^ (IPA^®^; build version 212183, content version 14855783 (release date 2013-02-05); Ingenuity Systems, Redwood City, CA, USA). In addition, IPA^®^ and its knowledge base were also used to generate biological networks reflecting gene-gene relationships.

#### Gene expression signature generation

A gene expression signature able to discriminate between spontaneously arising tumors and tumors that developed following cigarette smoke exposure was extracted from the interaction coefficient CeT:doH by using an in-house developed program, *GenSigPred*. For any gene expression profile T and phenotype G, the size of the gene expression signature, N, and the number of resamplings R were predefined. *GenSigPred* used a multistep process whereby the data set was randomly split into two parts, consisting of a training set with 20% of the samples and a test set with the remaining 80% samples. In the training data set, the genes were ranked based on statistics obtained by supervised machine-learning approaches including significance analysis of microarrays (SAM) (Tusher *et al.*, 2001) and the top N probe sets/genes GS(N, iter) in the rank list were selected. In the second step a classifier C(N, iter) was built based on the N genes by using a support vector machine (SVM) algorithm (Cortes & Vapnik, 1995) from the R package e1071. The predictions for every sample in the test set and the prediction performance P(N, iter) were recorded. The above procedure was performed R=50 times. The overall prediction error for specific N, Per(N) = Average(P(N,1), P(N,2), …, P(N,R)), was estimated. The union of genes was defined as GSA = {GS(N,1), GS(N,2), …, GS(N,R)}. The consensus of top genes in the R rank lists was selected based on the frequency of emergence of genes in the union of genes. Different values of N were tried and the value with good performance was selected.

#### MicroRNA microarray data preprocessing

Following the Hy3™-based “pseudo-single-color” pipeline used by Exiqon (Vedbaek, Denmark), the raw data were preprocessed by using the Bioconductor *limma* package in the R statistical computing environment (Smyth, 2004). A *normexp* background subtraction (*offset*=10) was first applied (Ritchie *et al.*, 2007) followed by a probe-level quantile normalization (Bolstad *et al.*, 2003). The probe set intensities were obtained by median summarization. All arrays satisfied the standard quality control criteria. However, one outlier was detected based on between-array correlations and was excluded from further analysis. The probe sets were then filtered for the mouse miRNA, “*present*” in at least half of the samples of at least one of the four sample groups. The “*present*”/”*absent*” calls were based on Exiqon's recommendation to take a detection threshold at the 5^th^ percentile of the distribution of the intensities measured in a given array. This yielded a final 260*70 miRNA expression matrix.

#### MicroRNA microarray data analysis

The data available for miRNA expression analysis are summarized in Table 2. In some cases, technical replicates were incorporated to obtain the final number of samples per experimental group. Increasing the number of samples to 10 in this fashion resulted in a decrease of the noise which was expected to occur from miRNA hybridization analysis.

**Table 2:** Summary of tissue samples used for miRNA expression analysis.

Tissue	Number of Mice			
	Sham	MS-75	MS-150	MS-300
Parenchyma	10	10	10	10
Tumor	8	7	6	9

Correlations between a small number of samples due to their provenance from the same animal were not taken into account and independence between all of the samples was assumed. This assumption enabled us to use the same linear model that was used for gene expression analysis (see Gene Expression Interaction Analysis). Here, the interaction coefficient *CeT:doH* signifies the difference in miRNA expression between tumor and parenchyma of the mice from the MS-300 group as compared to the difference in miRNA expression between tumor and parenchyma of sham controls. A less stringent FDR cut-off of 0.05 was applied.

## Results

### Histopathology

The results of the histopathologic evaluation of tumors from both sham control and MS-exposed A/J mice have been reported previously (Stinn *et al.*, 2013a). Briefly, adenomas presented the highest contribution to lung tumor incidence, followed by carcinomas and nodular hyperplasias, independent of exposure conditions (MS or sham). In addition, while the incidence of nodular hyperplasia was widely independent of MS exposure, there was a higher incidence of both adenomas and carcinomas in the cigarette-smoke-exposed animals, compared to animals in the sham control group. However, only adenoma incidence in the MS-150 and MS-300 groups was significantly different from that observed in the sham control animals (*p*<0.001).

### Gene expression analysis

**Figure 1:**
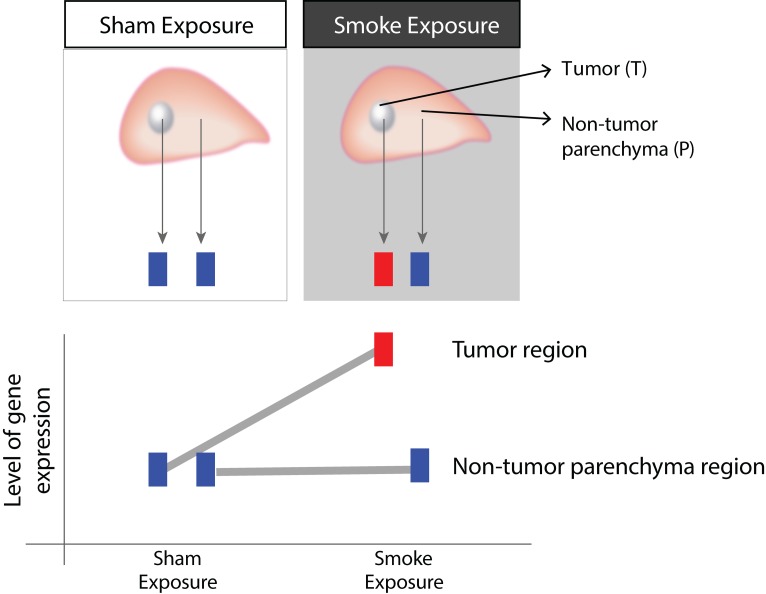
Illustration of the interaction analysis. The upper panel shows the two different tissue types (tumor (T) and non-tumorous parenchyma (P)) that were isolated from animals following sham and mainstream cigarette smoke (MS) exposure. Rectangles (blue or red) illustrate various expression levels of a gene. The lower panel depicts the interaction term reflecting the changes of gene expression which were different in T as compared to the surrounding P tissues following MS exposure. In other words, the genes with significant interaction are those whose levels were differentially affected between the two tissue types upon MS exposure.

We sought to identify genes that were differentially expressed in tumors of A/J mice exposed to cigarette smoke compared to tumors arising spontaneously and distinguished them from genes that were not affected by exposure in the parenchyma surrounding these tumors (Figure 1). Therefore, we applied an interaction term analysis and found the expression levels of 1, 2 and 269 genes to be statistically significantly altered between parenchyma and tumor tissues in the MS-75, MS-150, and MS-300 groups, respectively (FDR <0.01). Due to the few gene expression changes induced by lower MS concentrations, we focused on the biological interpretation of the results obtained from the *CeT:doH* interaction term analysis.

**Figure 2:**
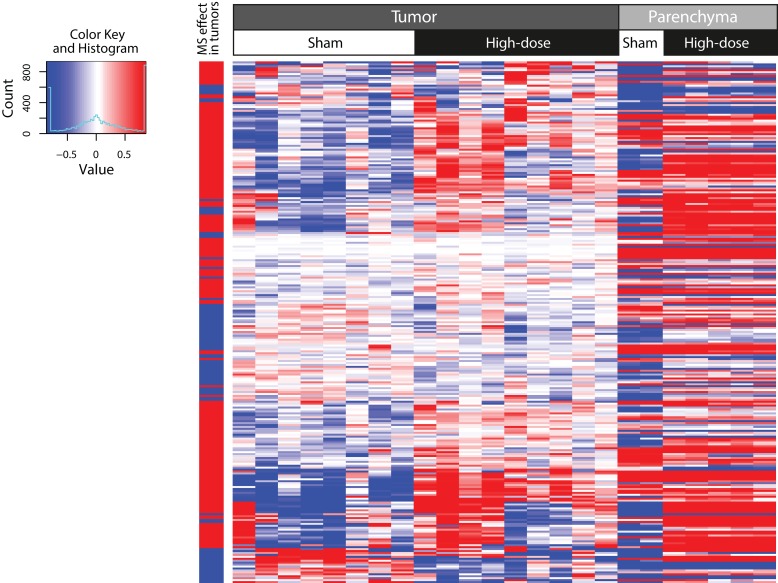
Two-dimensional hierarchical cluster of genes that were expressed in a different manner in tumor compared to surrounding, non-tumorous parenchyma following MS-300 exposure. Columns represent mRNA samples from different tissues and exposures as indicated in the label above the heatmap. Expression values of every gene (rows) are centered by their mean across all tumor samples. The column on the far left denotes the increase (red) or decrease (blue) in fold change in the tumor tissue in response to MS exposure. This heatmap was generated by using the hclust function in the “stats” R package with complete agglomeration and Euclidean distance metrics.

Hierarchical clustering showed that the up- and down-regulated genes clearly segregated according to the expression values by tissue (tumor vs. parenchyma) and exposure (sham vs. MS exposure; Figure 2).

**Figure 3:**
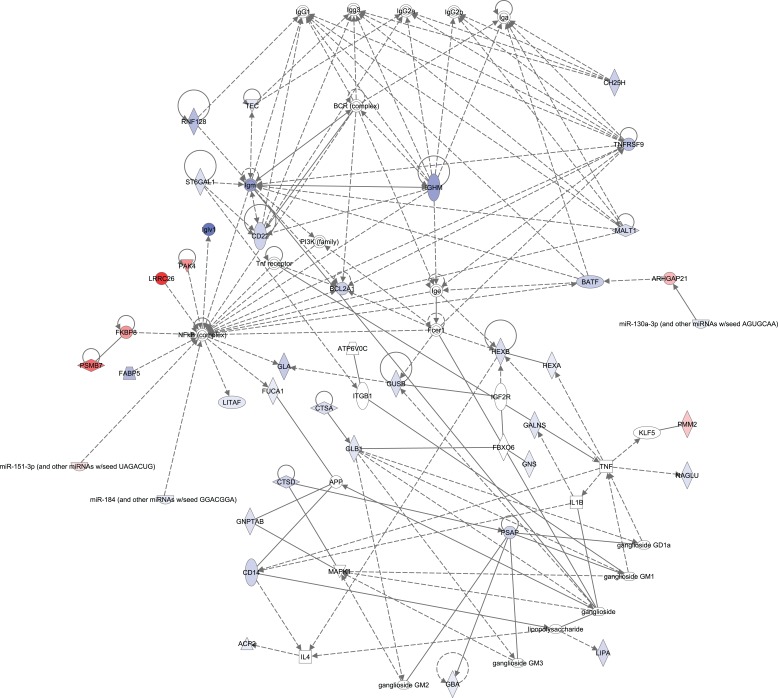
A biological network capturing the immune response and the metabolism of glycosaminoglycan and sphingolipid revealed from Ingenuity Pathway Analysis^®^ (IPA^®^). Molecules in the network represent the genes that were expressed in a different manner in tumor compared to surrounding non-tumorous parenchyma tissues following MS-300 exposure (significant interaction analysis). Blue and red indicate significantly decreased and increased fold change, respectively, of the gene expression affected by cigarette smoke in the tumors as compared to the parenchyma and sham controls. Color intensity qualitatively represents the degree of fold change. Straight and dashed lines specify direct and indirect interaction, respectively.

Analysis of the 269 differentially expressed genes using IPA^®^ further revealed a suppression of the humoral immune response in tumors from MS-exposed mice. Cigarette smoke exposure resulted in a more indolent immune response in the tumors compared to the parenchyma as indicated by the decrease in expression of genes contributing to the humoral immune response network (Figure 3). The IPA^®^ analysis further highlighted several biological functions associated with this network, including decreased immunoglobulins IgM and IgG as well as the reduction in B cell numbers. Consistent with these observations, the expression of several genes that are essential for the development and activation of B cells such as *Batf*, *Cd22*, *Ch25h*, *IghM*, *St6gal1*, and *Tnfrsf9* was found to be decreased or remained unaltered in tumor tissues of the MS-exposed mice, while their expression levels rose in the surrounding parenchyma.

One of the central genes in the network is *Nfkb,* which IPA^®^ predicted to be less altered (z-score –3.187) in tumors from MS-300 mice. Supporting this prediction are the downregulation of the immune response-related gene *IgIv1* and the fatty acid binding protein-encoding gene *Fabp5* downstream of *Nfkb*. Interestingly, despite the negative interaction coefficient for *Fabp5*, the *Fabp5* expression level in the tumors of sham mice was always higher compared to that in parenchyma, whereas it remained unaltered in parenchyma and tumor tissues from MS-300 animals. The network also shows inverse associations between the downregulation of *Nfkb* and upregulation of both *Lrrc26* and *Pak4* mRNA levels (Figure 3).

Furthermore, IPA^®^ highlighted a stronger perturbation of sphingolipid and glycosaminoglycan metabolism in tumor tissues than in parenchyma from MS-300 animals compared to sham controls (Figure 3). In particular, our data indicate enhanced accumulation of glycosphingolipids, glycosylceramide, glycosaminoglycans and lipids in cigarette smoke-related tumors, compared to spontaneous tumors (z-scores between 1.631 and 2.436), while processes contributing to cellular homeostasis of lipid metabolites, such as transport, efflux and degradation, appeared to be suppressed following exposure (z-scores between –1.038 and –2.176). This was linked to a global reduction in the expression of genes encoding lysosomal enzymes, including *Gla*, *Glb1*, *Gba*, *Gns*, *Galns*, *Gusb*, *Ctsa*, *Ctsd*, *Hexa*, and *Hex*, and the downregulation of the prosaposin (*Psap*) and *Atp6v0c* mRNA in tumors of A/J mice exposed to MS.

### Gene expression signature generation

**Figure 4:**
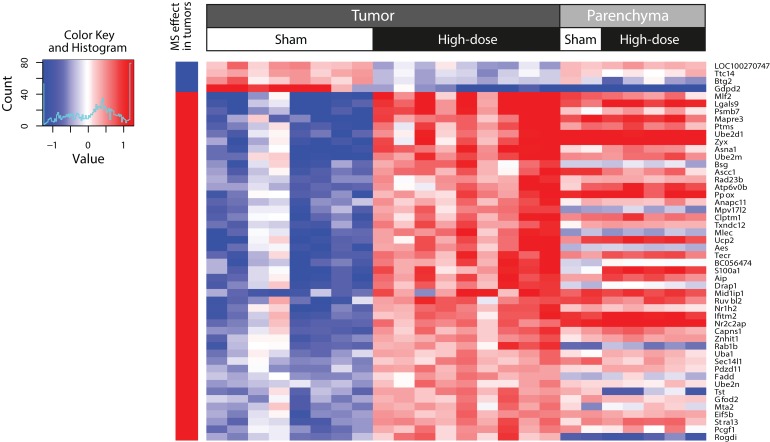
Two-dimensional hierarchical cluster of a gene signature that could discriminate between spontaneous tumors in sham controls and tumors from MS-300 mice. Columns represent samples from different tissues and exposures. Expression values of every gene (rows) are centered by their mean across all tumor samples. The column on the far left denotes the increase (red) or decrease (blue) in fold change in the tumor tissue in response to MS exposure. This heatmap was generated by using the hclust function in the “stats” R package with complete agglomeration and Euclidean distance metrics.

A gene expression signature consisting of the expression profiles of 50 genes was extracted from the interaction coefficient *CeT:doH*. This gene expression signature was able to discriminate between lung tumors spontaneously arising in the lungs of A/J mice exposed to air and lung tumors developing in the MS-300 A/J mice (Figure 4). Moreover, application of the gene signature to the entire tumor sample set demonstrated that it clearly separated the tumors into two groups. While one group (cluster 1) reflects the gene signature profiles of all sham control and MS-75 group samples, the other group (cluster 2) is primarily formed on the basis of the expression of the 50 genes of interest in all MS-150 and MS-300 group tumors (Figure 6). The corresponding heat map also provides evidence that the expression profiles of the 50 signature genes behave similarly in tumor tissues from sham control mice and from those in the MS-75 group, possibly reflecting a high similarity between the lung tumors from these groups, resulting from a lack of cigarette smoke effects. Interestingly, two tumors from A/J mice from the MS-150 group appeared to exhibit more similarities in gene signature expression to sham control and MS-75 group samples. This could indicate that those tumors developed spontaneously and have not been affected by MS exposure to the degree as the other tumors in the MS-150. However, further studies are necessary to corroborate this observation.

### MicroRNA expression

**Figure 5:**
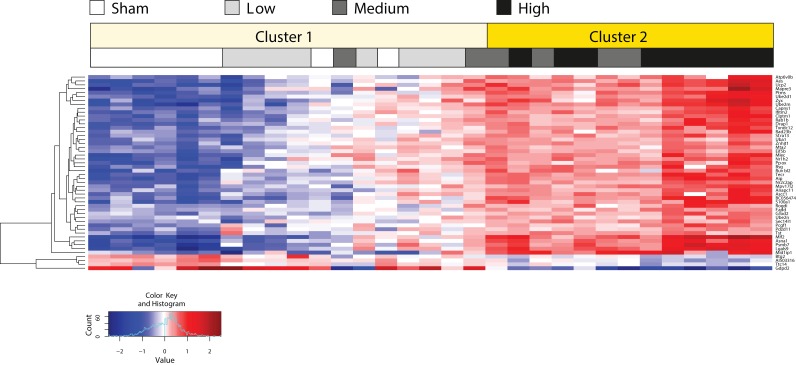
Two-dimensional hierarchical cluster of the miRNA expressed in a different manner in tumor compared to the parenchyma following MS-300 exposure. Rows correspond to the 70 miRNAs with statistically significant interaction coefficient (FDR<0.05, p-value<0.05). Columns represent miRNA samples from different tissues and exposure. The three columns on the far left indicate the specific miRNA that were altered by MS exposure specific to the tumor or surrounding, non-tumorous parenchyma tissues and those that were differently altered in the tumor compared to the surrounding non-tumorous parenchyma upon MS exposure (interaction analysis). This heatmap was generated using the hclust function in the “stats” R package with complete agglomeration and Euclidean distance metrics.

The differential effect of MS exposure on miRNA expression in A/J mouse tumors was also captured by the coefficient of the explanatory interaction variable in the linear statistical model described above. After performing the same interaction calculation used for gene expression analysis, we found 70 miRNA that were statistically significantly altered in the MS-300 group (FDR <0.05). Surprisingly, the effects of cigarette smoke on miRNA levels were greater in parenchymal tissues than in tumors (Figure 5 and 7), and miRNA were distributed across three major categories of unequal sizes: 62 mRNA were altered (raw *p*-values were smaller than 0.05 for the corresponding pairwise comparisons) in parenchyma only, 3 miRNA (mmu-miR-130a-3p, mmu-miR-151-3p, mmu-miR-184-3p) were altered in tumors only and 5 in both tumor and parenchyma. A closer look at those 5 miRNA revealed that the expression of 4 miRNA including mmu-miR-291a-5p, mmu-miR-291b-5p, mmu-miR-664-3p, and mmu-miR-1306-3p increased in tumor tissues following cigarette smoke exposure, yet they decreased in the parenchyma of exposed mice, whereas mmu-miR-378-3p displayed the same behavior in both tissues following MS exposure. The first and largest group of miRNA, displaying altered expression only in parenchyma seems *a priori* irrelevant since expression of these miRNA in tumors did not change following MS exposure. However, the dual interpretation of the interaction can be leveraged to help in understanding their behavior. We therefore considered the tumor *vs.* surrounding parenchyma tissue comparisons in the presence or absence of MS exposure. This allowed for a division of the 62 miRNA that changed only in parenchyma into further subgroups. For example, we found that the expression of 3 miRNA (mmu-miR-130b-3p, mmu-miR-135a-5p, mmu-miR-692) displayed no differences between tumor and parenchyma in the absence of cigarette smoke exposure and therefore their interaction statistics reflected exclusively the effects of exposure. In contrast, expression of 35 miRNA was not different between tumor and parenchyma in the presence of MS exposure. Thus their expression level in spontaneous tumors corresponds to their expression level in both parenchyma and tumor tissues from exposed mice. In this case, the cigarette smoke effect on parenchyma was the same as the (non-exposed) tissue effect (i.e.transformation of the parenchyma into tumor tissue). Finally, the remaining 24 miRNA exhibited differential expression between tumor and parenchyma in both the absence and presence of cigarette smoke exposure, including 2 miRNA (mmu-miR-135b-5p, mmu-miR-1198-5p), whose expression levels increased in parenchyma but remained stable in tumor tissue following MS exposure, 5 miRNA (mmu-miR-21-5p, mmu-miR-31-5p, mmu-miR-146b-5p, mmu-miR-665-3p, mmu-miR-744-5p) that remained more highly expressed in tumors compared to parenchyma, and 17 miRNA with lower expression levels in tumors than in parenchyma, even upon cigarette smoke exposure (Figure 8).

**Figure 6:**
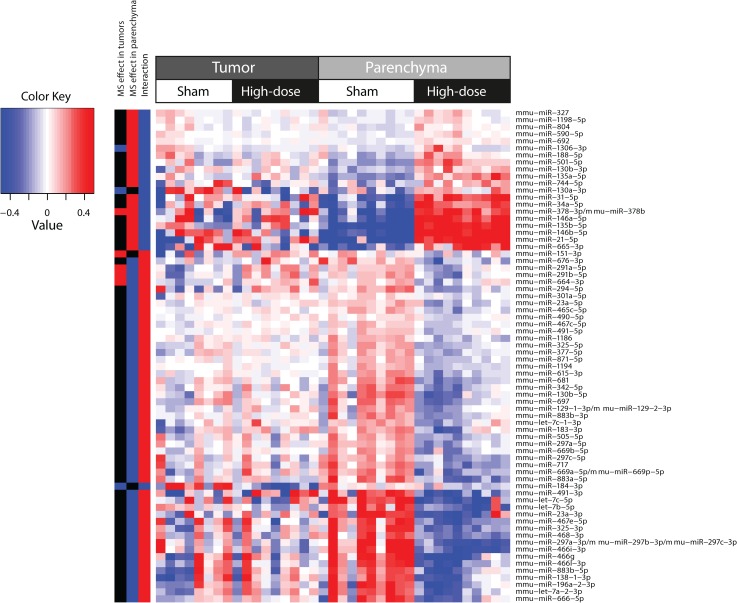
Hierarchical cluster based on the extracted gene signature and clustering applied to all samples. Expression values of every gene (rows) are centered by their mean across all tumor samples. Columns represent samples from different tissues and exposure. Two-way hierarchical clustering was performed with squared Euclidian distance metrics and complete agglomeration method.

**Figure 7:**
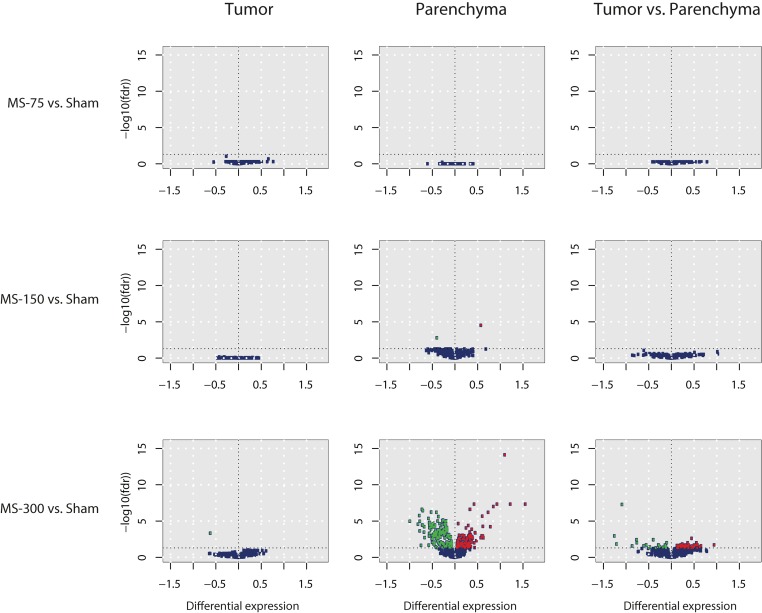
Volcano plots displaying the dose-dependent MS effect on miRNA expression. The three rows correspond to the three MS concentrations, the three columns to the relevant tissue comparisons. MicroRNA with FDR<0.05 are colored according to the sign of their differential expression, i.e., red for increased and green for decreased. The y-axis indicates -log10(FDR) and the x-axis indicates negative and positive differential expression of the miRNA.

**Figure 8:**
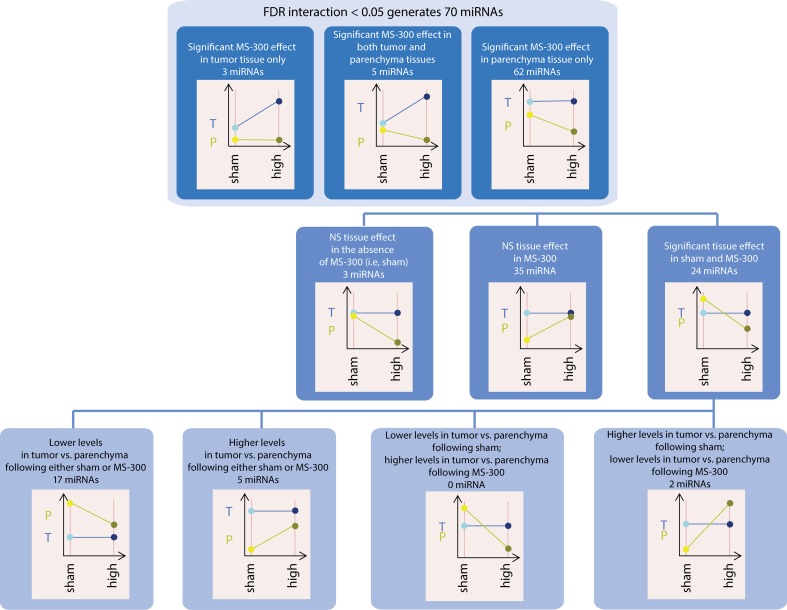
Tree representing the subset analysis of the 70 miRNA with statistically significant interaction coefficient (FDR<0.05). First, within the 70 miRNA, the MS effect was determined by the two relevant comparisons: the level of miRNA in MS-300 exposed tumor vs. sham control tumor and the level of miRNA in MS-300 exposed parenchyma vs. sham control parenchyma. Secondly, the 62 miRNA were further grouped based on the tissue effects in sham control and MS-300. Finally, the 24 miRNA with significant tissue effect were further grouped based on their levels in tumor and surrounding parenchyma following the sham and MS-300 exposure. In each case a graph shows a typical example of the corresponding interaction graph.

## Discussion

Although spontaneous lung tumors in mice are often similar in morphology, histopathology, and molecular characteristics to human adenocarcinomas (Meuwissen & Berns, 2005), mouse models of lung cancer still cannot fully reflect the complexities of human lung cancer (Dutt & Wong, 2006). The underlying mechanisms of tumorigenesis are not yet fully elucidated or clearly understood.

Our gene expression analysis of lung tumors arising in A/J mice exposed to air and to MS indicated that there was no noticeable humoral immune response within the MS-related tumors compared to the non-tumor parenchyma. The humoral immune response protects the extracellular spaces, in which B cells produce antibodies that aid in the destruction of extracellular invaders. The differentiation of B cells into antibody-secreting plasma cells upon recognition of a specific antigen usually requires T helper cells (CD4+ T cells) (Janeway *et al.*, 2001). It has previously been reported that cigarette smoking decreases dendritic and T cell numbers, as well as immunoglobulin IgG levels (Robbins *et al.*, 2004). Accordingly, we observed that MS exposure influenced genes regulating B cell activation and development in that the expression of these genes was greater in lung parenchyma compared to the tumor tissue. This finding supports a study by Chew and colleagues (2010) who observed that the expression of genes regulating various immune cell types and functions was more enhanced in the adjacent non-tumor tissues compared to the tumor samples from 68 patients with hepatic cellular carcinoma (Chew *et al.*, 2010). Furthermore, the predicted downregulation of *Nfkb*, which contributes to the maturation of T and B cells particularly during the late stages of their development, also suggests that other key players of the immune response might be negatively affected by cigarette smoke exposure. For example, the observed reduced expression of *Fabp5* downstream of *Nfkb*, one of the genes defining the profile of resting tumor-associated macrophages (Biswas *et al.*, 2006), might be an indication of a tumor microenvironment that supports rather than inhibits tumor growth in mice exposed to cigarette smoke (Redente *et al.*, 2010). On the other hand, *Nfkb* activation could be suppressed in the lung tumors of MS-exposed mice due to the overexpression of *Lrrc26,* which has also been shown to occur in the breast cancer cell line MDA-231 and the epidermoid cancer cell line A431 (Liu *et al.*, 2012). Not only did the overexpression of *LRRC26* result in a reduction of *NFkB* mRNA levels in MDA-231 cells, it also resulted in the suppression of constitutive NFkB activation and negative regulation of chemokine and cytokine genes and contributed to a tumor microenvironment rich in extracellular matrix components in xenograft models (Liu *et al.*, 2012). Moreover, the inverse association between the downregulation of *Nfkb* and upregulation of *Pak4* is consistent with a previous observation in which knockdown of *PAK4* by RNA interference resulted in activation of NFkB in HeLa cells and increased expression of *PAK4* was associated with oncogenic transformation by suppressing apoptosis and promoting cell survival (Li & Minden, 2005).

Interestingly, of the 3 miRNA that were affected by cigarette smoke exposure in tumors only, mmu-miR-151-3p, mmu-miR-130a-3p and mmu-miR-184-3p have previously been implicated in carcinogenesis. MicroRNA-151a, whose expression levels in lung tumors of MS-300 A/J mice was higher than in spontaneous tumors, has been reported to be induced in a variety of solid cancers (McNally *et al.*, 2013; Gu *et al.*, 2013). Further, reduced miR-130a expression has been observed in non-small cell lung carcinoma (NSCLC) cell lines (Acunzo *et al.*, 2012) and squamous cell carcinomas of the lung (Gao *et al.*, 2011). Similar to our observation of MS-exposure-related suppression in A/J lung tumors, miR-130a expression was also found to be down-regulated in airway epithelial cells of healthy smokers compared to levels observed in non-smokers (Schembri *et al.*, 2009) and in lung organotypical cultures exposed to MS (Mathis *et al.*, 2013). These findings suggest that miR-130a transcription in the lung is modulated by cigarette smoke exposure and that deregulation of its activity may contribute to smoking-induced lung disease.

Finally, we found mmu-miRNA-184 expression to be suppressed in response to MS. High expression of miR-184 has been reported in squamous cell carcinomas of the tongue, and when expression of the mature miRNA was knocked down by RNA interference, tongue cancer cells proliferated less and became increasingly apoptotic (Wong *et al.*, 2008). Conversely, overexpression of miR-184 in neuroblastoma cell lines resulted in a significant inhibition of tumor cell proliferation and reduction of tumor growth in a xenograft model by induction of apoptosis (Foley *et al.*, 2010), leading us to speculate that apoptosis in the lung tumors of exposed mice might be inhibited. Taken together, these results suggest that cigarette smoke exposure alters the tumor microenvironment and affects the regulation of miRNA with potential oncogenic properties, thus potentially contributing to tumor development and progression in this mouse model of smoking-related lung tumorigenesis.

Interestingly, we also found supporting evidence for perturbed glycosphingolipid and glycosylceramide degradation or lysosomal dysfunction in lung tumors from MS-exposed mice when compared to spontaneously arising tumors. Sphingolipids are essential plasma membrane components and degradation of sphingolipids, a complex process which is regulated by the synthesis and degradation of the sphingolipids themselves, takes place in the lysosome. There, lysosomal hydrolases facilitate the degradation of intra- and extracellular macromolecules such as proteins, DNA, RNA, polysaccharides, and lipids following endocytosis, phagocytosis or as part of autophagic recycling. However, the data reported here are not in support of activated autophagy, which was previously shown to increase in association with cigarette smoke-induced lung injury in mice (An *et al.*, 2012) and men (Zhu *et al.*, 2013). IPA^®^ showed that the expression of lysosomal regulators such as *Igfr2* remained unchanged, suggesting that lysosomal trafficking might not be transcriptionally deregulated. Surprisingly, in tumors of A/J mice exposed to MS, a global reduction in the expression of genes encoding lysosomal enzymes was seen, suggesting that glycosphingolipid degradation was hampered by lack of lysosomal enzymes. In addition, the observed decrease in *Psap* gene expression could also explain the accumulation of glycosphingolipids, which may have ultimately resulted in lysosomal dysfunction (Schulze *et al.*, 2009; Tatti *et al.*, 2011). Moreover, suppression of *Atp6v0c* gene expression could point to the prevention of lysosomal leakage which releases breakdown products for reuse (Byun *et al.*, 2011). Lysosomal leakage is also known to induce cell death, by apoptosis or necrosis, dependent on the extent of lysosomal damage (Yamashima & Oikawa, 2009). Suppression of this event would explain why downstream apoptotic pathways remain largely unaffected – at least on the transcriptional level. All these data suggest that exposure to MS disrupts physiological lysosome function by interfering with the hydrolysis of glycosphingolipids, glycosylceramide and glycosaminoglycans, thus preventing the execution of autophagic and/or apoptotic programs in lung tumor cells in A/J mice exposed to MS.

Our findings further indicate an intricate interlinking of lysosomal dysfunction and perturbations of the anti-tumor immune response following exposure to cigarette smoke considering the noticeable overlap between the humoral immune network and the activated biological functions pertaining to sphingolipid and glycosaminoglycan metabolism in our data set. Faulty degradation and subsequent accumulation of glycosphingolipids have been associated with systemic effects such as an increased pro-oxidant and pro-inflammatory state (Xu *et al.*, 2010). Glycosphingolipids have also been proposed to facilitate the interaction of immune cells with endothelial cells and to contribute to natural killer T (NKT) cell-mediated immune responses (Schnaar *et al.*, 2009). NKT cells are a heterogeneous group of T cells that recognize antigens presented by CD1d non-MHC antigen-presenting molecules. In mice, CD1d loading occurs in the lysosomal compartment, and this process is suspected to be aided by saponins (Zhou *et al.*, 2004). NKT cells that recognize CD1d-presented glycolipid complexes have been implicated in various immunoregulatory functions including the assistance in B cell activation and the mounting of an antibody-specific response (Galli *et al.*, 2007). Based on the IPA^®^-predicted reduction in B cell function and activation and the apparent lysosomal dysfunction we observed, the overall suppression of the humoral immune response and apparent lack of glycosphingolipid and glycosylceramide turnover in tumors from MS-exposed A/J mice could be linked with insufficient antigen presentation potentiating the ability of tumor cells to escape from immune surveillance in a “primed”, tumor-friendly environment.

The widely described finding that glycosphingolipids are expressed on human tumor cells and function as modulators of immune mechanisms in human cancers can be exploited for therapeutic purposes (Durrant *et al.*, 2012). However, to our knowledge, this is the first study reporting the interplay between the anti-tumor immune response and glycosphingolipid metabolism in a mouse model of lung cancer, and further studies are required to explore this connection in more detail. This is also true for the substantiation of the gene signature we established as a potential discriminator between spontaneously arising and cigarette smoke-related lung tumors in A/J mice. Eventually, additional supporting data might provide a basis for extrapolating results from *in vivo* carcinogenicity studies to assessing lung cancer risk in smokers in the future.
